# Period and cohort effects: consequences on spirometric lung function in Norway during the 20th century

**DOI:** 10.1183/23120541.00302-2022

**Published:** 2022-12-12

**Authors:** Lucia Cestelli, Ane Johannessen, Knut Stavem, Amund Gulsvik, Rune Nielsen

**Affiliations:** 1 Department of Clinical Science, University of Bergen, Bergen, Norway; 2 Centre for International Health, Department of Global Public Health and Primary Care, University of Bergen, Bergen, Norway; 3 Pulmonary Department, Akershus University Hospital, Lørenskog, Norway; 4 Institute of Clinical Medicine, University of Oslo, Oslo, Norway; 5 Health Services Research Unit, Akershus University Hospital, Lørenskog, Norway; 6 Department of Thoracic Medicine, Haukeland University Hospital, Bergen, Norway

## Abstract

**Background and aim:**

Several factors can influence measured lung function over time. The aim of this study was to investigate period and cohort effects on spirometric measures in a large general population sample in Norway during the 20th century, using Global Lung Function Initiative (GLI-2012) equations as a reference.

**Methods:**

36 466 subjects (born 1894–1969) from four cross-sectional surveys conducted between 1965 and 1999 were included, with harmonised data on smoking habits, respiratory symptoms, lung diseases, education and spirometry. Changes in forced expiratory volume in 1 s (FEV_1_) and forced vital capacity (FVC) z-scores in healthy subjects across surveys were explored to investigate period effects. Linear mixed-effects models of FEV_1_ and FVC z-scores on birth cohort, with survey as random effect, were used to investigate cohort effects, both in subjects of the total population and in healthy ones.

**Results:**

Relatively higher FEV_1_ and FVC z-scores in healthy subjects were found in the first survey (1965–1970) compared to the more recent ones (1988–1999), suggesting period effects. FEV_1_ and FVC z-scores increased significantly with birth cohort from 1894 to 1935, after adjustment for covariates. A more stable trend of FEV_1_ and FVC z-scores with birth cohort was evidenced for subjects born more recently (1945–1969).

**Conclusions:**

An increase of lung function with year of birth was observed in Norwegian subjects during the first half of the 20th century. The impact of period effects on lung function decreased from 1965 to 1999.

## Abstract 2

**Both period and cohort effects were observed in this study of lung function in Norwegian subjects during the 20th century. FEV_1_ and FVC increased during this time as a result of cohort effects.** https://bit.ly/3yj3i8L

## Introduction

The measurement of lung function is fundamental for the diagnosis and management of several respiratory diseases. Numerous studies have described trends in prevalence of chronic respiratory diseases worldwide and in Norway in different periods of time [1–6], but changes in lung function over the past century have not been fully elucidated.

Different factors may influence lung function across periods of time. These factors can be characterised as period and cohort effects. Period effects are changes dependent on the time of measurement and affect all ages and cohorts within that time of measurement simultaneously [7]. Cohort effects are changes related to different exposures that characterise populations from the same period of birth [7].

Improvements in spirometry methodology may contribute to the variability of lung function measured at different points in time as period effects. Temporal changes in smoking habits, nutrition, socioeconomic status and environmental exposures, on the other hand, may affect lung function as cohort effects.

Secular trends in spirometric data during the 20th century have been described previously in different populations [8–12]; however, most of the studies explored only cohort and not period effects.

The Norwegian Population Survey Initiative on Respiratory Health in Adults (NPSIR) is a project focusing on the study of chronic inflammatory respiratory diseases and their determinants in Norwegian adults [13]. In total, random population samples of about 178 000 subjects in the counties of Oslo, Hordaland and Rogaland were invited in seven cross-sectional surveys, conducted at different time points – the first was conducted in 1965.

We hypothesised that in the Norwegian population during the 20th century measures of lung function have changed both in relation to year of measurement (period effects) and year of birth (cohort effects). We therefore aimed to investigate the presence of both changes in over 35 000 subjects born 1894–1969 participating in four surveys of NPSIR, using spirometric measures and Global Lung Function Initiative (GLI-2012) equations as reference.

## Methods

### Study population

The study population consists of pooled data from four cross-sectional surveys that are part of NPSIR: the Bergen Chronic Respiratory Disease Survey (BCRDS, 1965–1970), the Pneumoconiosis Survey of Western Norway (PSWON, 1988–1990), the Hordaland County Respiratory Health Survey (HCRHS, 1996–1997) and the Bronchodilatation Survey of the Hordaland Health Study (BD-HUSK, 1998–1999). The characteristics of the four surveys have previously been described in detail [13] and are summarised in supplementary table S1. The study was approved by the Committee on Medical Research Ethics (2017/1679), The Norwegian Data Inspectorate (07/00414) and The Norwegian Directorate of Health (07/948).

### Questionnaires

All surveys used standardised questionnaires including information on smoking habits, respiratory symptoms and lung diseases. The first survey used an interview questionnaire, while the following surveys used self-administered questionnaires. Smoking status was categorised as never, former or current. Smokers were defined as subjects smoking cigarettes, pipe, cigars or a combination of them. Pack-years were calculated as the number of packs of cigarettes smoked daily multiplied by years of smoking. For hand-rolled cigarettes the equivalence 1 g tobacco=1 cigarette was used. The respiratory symptoms selected for the analysis were: breathlessness walking uphill and/or climbing two flights of stairs, attacks of breathlessness, morning cough and chronic cough. Information regarding the presence of lung diseases (asthma, bronchitis, emphysema, COPD) was also collected.

### Education

Education data were retrieved from the registers of Statistics Norway, which provided the highest attained education for each decade for each subject based on national census data in 1970, 1980, 1990 and 2001 [14, 15]. Data were converted into number of years of education and grouped into three categories (compulsory education <11 years, medium level 11–13 years, university level 14–20+ years).

### Lung function

Pre-bronchodilator spirometry with measurement of absolute values of forced expiratory volume in 1 s (FEV_1_) and forced vital capacity (FVC) was performed in all surveys. A Vitalograph wedge bellows dry spirometer (Vitalograph Ltd., Buckingham, UK) (P-model or S-model) was used in BCRDS, PSWON and BD-HUSK, and a Gould 2100 mass flow anemometer (Gould Electronics BV Medical Products, Bilthoven, The Netherlands) was used in HCRHS. Spirometry was performed according to the guidelines valid at the time of the survey in PSWON (1988–1990), HCRHS (1996–1997) and BD-HUSK (1998–1999) [16, 17]. At the time of the first survey (BCRDS, 1965–1970) no international recommendations were available, and the best results of two acceptable manoeuvres were collected [18]. Measurements were recorded at body temperature and pressure saturated conditions (BTPS) in all surveys, except for BCRDS, where they were recorded at ambient temperature pressure saturated conditions (ATPS) and converted to BTPS by a correction factor. Z-scores, a measure of how many standard deviations the value is from its predicted, were calculated for FEV_1_ and FVC using GLI-2012 reference equations [19].

### Harmonisation

Individual-level data were harmonised across surveys. Subjects who participated in more than one survey were only included in their first survey. Missing values were <1% for smoking status and <2% for education and symptoms/lung diseases. All other variables were complete. Missing values were not imputed.

### Healthy subgroup

A selected subgroup of healthy individuals was identified to further analyse lung function in subjects who are traditionally selected for the development of reference equations. Subjects were defined as “healthy” if satisfying the following criteria: never-smokers, no obstructive lung diseases, no breathlessness walking uphill and/or climbing two flights of stairs, no attacks of breathlessness, no morning cough and no chronic cough.

### Statistical analysis

Population characteristics were described using measures of central tendency or distribution, as appropriate. To investigate the presence of period effects we examined the distribution of mean FEV_1_ and FVC z-scores in healthy subjects across surveys. Linear mixed-effects models of FEV_1_ and FVC z-scores on year of birth were used to investigate the presence of cohort effects. Year of birth was categorised into 10-year birth cohorts in order to explore potential nonlinear trends. Analyses were conducted stratified by sex. The following covariates were selected *a priori*: smoking status, pack-years, education, lung diseases and respiratory symptoms. Age and height were accounted for through the use of z-scores. Survey was included as random intercept effect to account for potential differences in lung function among surveys due to period effects. The models were developed in the total population, and results were presented for both subjects of the total population and healthy ones. In order to explore the potential contribution of height to the changes in lung function, we also developed models of height on birth cohort, adjusted for age, separately by sex. All analyses were conducted using Stata 17 (StataCorp LLC, College Station, TX, USA).

## Results

### Characteristics of the study population

The participation rates of the original surveys were 84% for BCRDS, 68% for PSWON, 75% for HCRHS and 69% for BD-HUSK. The final dataset after harmonisation of the four original cohorts consisted of 36 466 subjects (table 1). 30 531 (84%) subjects were males, of which 26 092 originated from the PSWON survey. Males were on average younger than females (mean age 40.4 *versus* 52.3). The year of birth of participants ranged from 1894 to 1969, and the study period ranged from 1965 to 1999. Among males, the number of current smokers was very high in the first survey and declined markedly over time, while variation in female smoking habits were more modest. The percentage of subjects with medium- and high-level education increased over time in both sexes. The variability of lung function reflected population demographics, with higher values in younger subjects and in males compared to females. Depending on the survey, 26–47% of subjects reported at least one respiratory symptom and 5–27% presence of lung diseases, with higher values in the two more recent examinations (supplementary table S2).

**TABLE 1:** Characteristics and spirometry values of participants in the study population, stratified by sex and by survey

	**BCRDS**	**PSWON**	**HCRHS**	**BD-HUSK**	**Total**
**Survey years**	1965–1970	1988–1990	1996–1997	1998–1999	1965–1999
**Birth years**	1894–1943	1944–1958	1914–1969	1925–1951^#^	1894–1969
**Number of subjects**	5616	26 092	1993	2765	36 466
**Males**					
Subjects	2524 (45)	26 092 (100)	814 (41)	1101 (40)	30 531 (84)
Age years	47.3±13.9	38.2±4.3	50.5±16.0	68.3±9.3	40.4±9.0
Height cm	174.6±6.6	178.8±6.3	177.5±6.9	175.7±6.6	178.3±6.5
Smoking status					
Nonsmoker	392 (16)	8133 (31)	267 (33)	247 (23)	9039 (30)
Ex-smoker	461 (18)	6395 (25)	274 (34)	640 (59)	7770 (26)
Current smoker	1671 (66)	11 488 (44)	273 (34)	206 (19)	13 638 (45)
Pack-years	13.8±13.8	16.1±9.4	17.4±14.7	20.9±18.1	16.1±10.6
Education					
<11 years	982 (41)	3476 (14)	158 (20)	229 (21)	4845 (16)
11–13 years	1110 (47)	15 450 (60)	459 (57)	550 (50)	17 569 (59)
14–20+ years	292 (12)	6785 (26)	189 (23)	311 (29)	7577 (25)
FEV_1_, L	3.88±1.05	4.28±0.62	3.68±0.95	2.90±0.74	4.18±0.74
FEV_1_, z-score	0.08±1.34	−0.08±0.96	−0.37±1.10	−0.55±1.10	−0.09±1.02
FVC, L	4.90±1.12	5.40±0.76	4.73±1.06	3.87±0.82	5.29±0.87
FVC, z-score	0.13±1.14	0.04±0.90	−0.26±0.95	−0.52±0.97	0.02±0.93
**Females**					
Subjects	3092 (55)	-	1179 (59)	1664 (60)	5935 (16)
Age years	48.3±14.0	-	50.6±15.0	60.8±12.2	52.3±14.8
Height cm	161.2±6.0	-	164.7±6.2	163.3±6.3	162.5±6.3
Smoking status					
Nonsmoker	1925 (62)	-	533 (45)	782 (48)	3240 (55)
Ex-smoker	144 (5)	-	274 (23)	454 (28)	872 (15)
Current smoker	1023 (33)	-	372 (32)	396 (24)	1791 (30)
Pack-years	6.6±6.2	-	11.9±10.1	13.3±11.0	9.9±9.4
Education					
<11 years	1717 (58)	-	264 (23)	438 (27)	2419 (42)
11–13 years	1062 (36)	-	649 (56)	862 (53)	2573 (44)
14–20+ years	198 (7)	-	253 (22)	339 (21)	790 (14)
FEV_1_, L	2.78±0.73	-	2.74±0.67	2.36±0.60	2.66±0.71
FEV_1_, z-score	0.10±1.29	-	−0.20±1.04	−0.37±1.06	−0.09±1.20
FVC, L	3.38±0.79	-	3.43±0.76	3.00±0.69	3.28±0.78
FVC, z-score	−0.02±1.13	-	−0.16±0.93	−0.38±0.92	−0.15±1.05

Variables are presented as mean±sd or n (%) as appropriate. Z-scores calculated according to Global Lung Function Initiative (GLI-2012) equations. BCRDS: Bergen Chronic Respiratory Disease Survey; PSWON: Pneumoconiosis Survey of Western Norway; HCRHS: Hordaland County Respiratory Health Survey; BD-HUSK: Bronchodilatation Survey of the Hordaland Health Study; FEV_1_: forced expiratory volume in 1 s; FVC: forced vital capacity. ^#^: two birth cohorts: 1925–1927 and 1950–1951.

In total, 8042 subjects (22% of the original population) met criteria for inclusion in the healthy subgroup (table 2).

**TABLE 2:** Characteristics and spirometry values of participants in the healthy subgroup, stratified by sex and by survey

	**BCRDS**	**PSWON**	**HCRHS**	**BD-HUSK**	**Total**
**Survey years**	1965–1970	1988–1990	1996–1997	1998–1999	1965–1999
**Birth years**	1894–1943	1944–1958	1914–1969	1925–1951^#^	1894–1969
**Number of subjects** **^¶^**	1438 (26)	5674 (22)	409 (21)	521 (19)	8042 (22)
**Males**					
Subjects	328 (23)	5674 (100)	154 (38)	136 (26)	6292 (78)
Age years	40.8±14.4	37.8±4.4	44.8±14.9	64.5±11.5	38.7±7.3
Height cm	175.5±6.6	179.3±6.3	179.4±6.8	176.5±6.5	179.0±6.4
Education					
<11 years	81 (26)	405 (7)	11 (7)	16 (12)	513 (8)
11–13 years	149 (47)	2922 (52)	77 (50)	60 (44)	3208 (52)
14–20+ years	87 (27)	2277 (41)	65 (42)	59 (44)	2488 (40)
FEV_1_, L	4.43±0.92	4.42±0.59	4.14±0.77	3.38±0.61	4.39±0.64
FEV_1_, z-score	0.71±1.11	0.11±0.92	−0.01±0.88	0.06±0.91	0.14±0.94
FVC, L	5.35±1.04	5.50±0.76	5.18±0.94	4.32±0.77	5.46±0.80
FVC, z-score	0.48±1.00	0.12±0.89	−0.03±0.81	−0.09±0.84	0.13±0.90
**Females**					
Subjects	1110 (77)	-	255 (62)	385 (74)	1750 (22)
Age years	47.6±14.1	-	50.7±15.2	61.7±12.3	51.1±15.0
Height cm	161.5±6.1	-	164.8±6.2	163.0±6.3	162.3±6.3
Education					
<11 years	559 (52)	-	48 (19)	89 (23)	696 (41)
11–13 years	420 (39)	-	118 (47)	194 (51)	732 (43)
14–20+ years	101 (9)	-	86 (34)	98 (26)	285 (17)
FEV_1_, L	2.91±0.68	-	2.87±0.64	2.47±0.55	2.81±0.67
FEV_1_, z-score	0.39±1.16	-	0.14±0.90	0.01±0.91	0.27±1.09
FVC, L	3.50±0.73	-	3.55±0.76	3.09±0.65	3.42±0.74
FVC, z-score	0.19±1.03	-	0.10±0.84	−0.11±0.83	0.11±0.97

Variables are presented as mean±sd or n (%) as appropriate. Z-scores calculated according to Global Lung Function Initiative (GLI-2012) equations. BCRDS: Bergen Chronic Respiratory Disease Survey; PSWON: Pneumoconiosis Survey of Western Norway; HCRHS: Hordaland County Respiratory Health Survey; BD-HUSK: Bronchodilatation Survey of the Hordaland Health Study; FEV_1_: forced expiratory volume in 1 s; FVC: forced vital capacity. ^#^: two birth cohorts: 1925–1927 and 1950–1951; ^¶^: percentage refers to the original population.

### Lung function and different surveys: period effects

**FIGURE 1:**
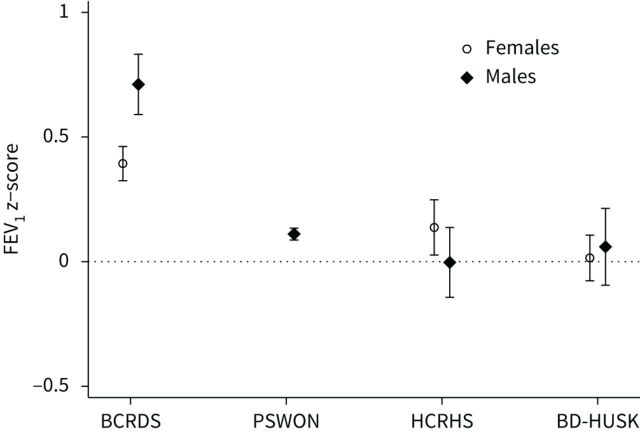
Mean forced expiratory volume in 1 s (FEV_1_) z-scores with 95% confidence intervals of healthy subjects in surveys conducted in different periods of time. Z-scores calculated according to Global Lung Function Initiative (GLI-2012) equations. BCRDS: Bergen Chronic Respiratory Disease Survey (1965–1970); PSWON: Pneumoconiosis Survey of Western Norway (1988–1990); HCRHS: Hordaland County Respiratory Health Survey (1996–1997); BD-HUSK: Bronchodilatation Survey of the Hordaland Health Study (1998–1999).

**FIGURE 2:**
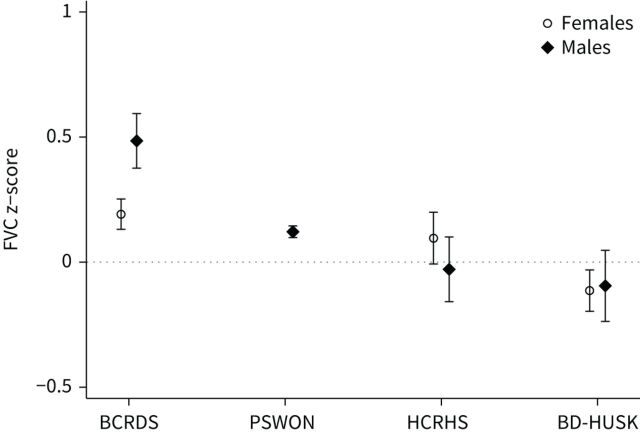
Mean forced vital capacity (FVC) z-scores with 95% confidence intervals of healthy subjects in surveys conducted in different periods of time. Z-scores calculated according to Global Lung Function Initiative (GLI-2012) equations. BCRDS: Bergen Chronic Respiratory Disease Survey (1965–1970); PSWON: Pneumoconiosis Survey of Western Norway (1988–1990); HCRHS: Hordaland County Respiratory Health Survey (1996–1997); BD-HUSK: Bronchodilatation Survey of the Hordaland Health Study (1998–1999).

Figures 1 and 2 illustrate the distribution of mean FEV_1_ and FVC z-scores in healthy subjects across surveys. FEV_1_ z-scores were close to zero in the three more recent surveys (1988–1999), but higher in the first one (1965–1970), especially in males, suggesting the presence of period effects. A similar trend was observed for FVC, though less evident.

### Lung function and year of birth: cohort effects

FEV_1_ and FVC z-scores increased with subsequent birth cohorts from 1894 to 1969, both in males and females, after adjustment for smoking habits, education, lung diseases and respiratory symptoms (tables 3 and 4). The pattern was however nonlinear, with a marked increase for birth cohorts from 1894 to 1935, followed by a more stable trend thereafter (1945–1969).

**TABLE 3:** Estimated changes of forced expiratory volume in 1 s z-scores with birth cohort in the total population

	**Males**				**Females**			
**Birth cohort**	**n**	**Coefficient**	**95% CI**	**p-value**	**n**	**Coefficient**	**95% CI**	**p-value**
**1894–1904**	469	-	-	-	640	-	-	-
**1905–1914**	517	−0.05	(−0.18–0.08)	0.440	720	0.19	(0.06–0.31)	<0.001
**1915–1924**	674	0.19	(0.07–0.31)	<0.001	790	0.31	(0.19–0.43)	<0.001
**1925–1934**	1552	0.51	(0.39–0.63)	<0.001	1580	0.61	(0.49–0.74)	<0.001
**1935–1944**	2125	0.74	(0.61–0.86)	<0.001	720	0.66	(0.53–0.79)	<0.001
**1945–1954**	17 838	0.66	(0.54–0.79)	<0.001	1095	0.57	(0.42–0.73)	<0.001
**1955–1964**	7223	0.59	(0.46–0.72)	<0.001	250	0.48	(0.28–0.68)	<0.001
**1965–1969**	133	0.59	(0.37–0.80)	<0.001	140	0.55	(0.32–0.79)	<0.001

Linear mixed-effect models with random effects at the survey level, adjusted for smoking status, pack-years, education, lung diseases and respiratory symptoms. Birth cohort reference category = 1894–1904. Z-scores calculated according to Global Lung Function Initiative (GLI-2012) equations.

**TABLE 4:** Estimated changes of forced vital capacity z-scores with birth cohort in the total population

	**Males**				**Females**			
**Birth cohort**	**n**	**Coefficient**	**95% CI**	**p-value**	**n**	**Coefficient**	**95% CI**	**p-value**
**1894–1904**	469	-	-	-	640	-	-	-
**1905–1914**	517	0.00	(−0.12–0.12)	0.990	720	0.13	(0.02–0.24)	0.020
**1915–1924**	674	0.14	(0.03–0.26)	0.010	790	0.28	(0.18–0.39)	<0.001
**1925–1934**	1552	0.43	(0.32–0.54)	<0.001	1580	0.54	(0.43–0.66)	<0.001
**1935–1944**	2125	0.61	(0.50–0.73)	<0.001	720	0.53	(0.41–0.65)	<0.001
**1945–1954**	17 838	0.60	(0.48–0.72)	<0.001	1095	0.50	(0.36–0.64)	<0.001
**1955–1964**	7223	0.61	(0.49–0.73)	<0.001	250	0.50	(0.32–0.68)	<0.001
**1965–1969**	133	0.70	(0.50–0.90)	<0.001	140	0.64	(0.43–0.85)	<0.001

Linear mixed-effect models with random effects at the survey level, adjusted for smoking status, pack-years, education, lung diseases and respiratory symptoms. Birth cohort reference category = 1894–1904. Z-scores calculated according to Global Lung Function Initiative (GLI-2012) equations.

**FIGURE 3:**
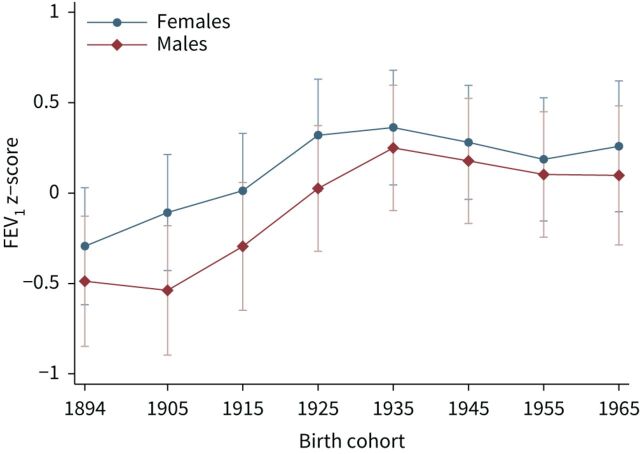
Adjusted predictions of forced expiratory volume in 1 s (FEV_1_) z-scores with 95% confidence intervals according to birth cohort, for subjects without a history of smoking, lung diseases and respiratory symptoms. Z-scores calculated according to Global Lung Function Initiative (GLI-2012) equations. Birth cohorts: 1894–1904, 1905–1914, 1915–1924, 1925–1934, 1935–1944, 1945–1954, 1955–1964, 1965–1969.

**FIGURE 4:**
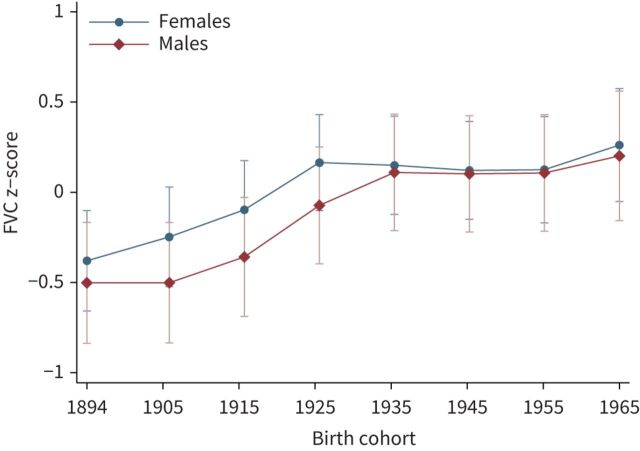
Adjusted predictions of forced vital capacity (FVC) z-scores with 95% confidence intervals according to birth cohort, for subjects without a history of smoking, lung diseases and respiratory symptoms. Z-scores calculated according to Global Lung Function Initiative (GLI-2012) equations. Birth cohorts: 1894–1904, 1905–1914, 1915–1924, 1925–1934, 1935–1944, 1945–1954, 1955–1964, 1965–1969.

Figures 3 and 4 illustrate the estimated changes of FEV_1_ and FVC z-scores with birth cohort in healthy subjects. Compared to predictions from the GLI-2012 equations, both males and females born approximately between 1894 and 1935 showed lower lung function values, with a rising trend over time. FEV_1_ and FVC z-scores of subjects born after 1935 were instead higher than GLI-predicted, without major changes depending on year of birth. Corresponding graphs for the total population showed relatively lower lung function values, but the overall pattern was very similar (supplementary figures S1 and S2).

Supplementary figure S3 illustrates the estimated changes of height according to birth cohort in the total population, for 45-year-old males and females. Both sexes showed a significant increase in height, especially until 1945, reflecting quite closely the spirometric measures.

## Discussion

We have found that during the 20th century, lung function in Norwegian general population samples increased with year of birth, both for males and females, after adjustment for several covariates. Our data also showed the presence of period effects in relation to the first survey included in our study, but not the more recent ones.

The presence of cohort effects in lung function has been reported previously. Xu *et al*. [8] showed an increase in height-adjusted FEV_1_ from older to younger birth cohorts in general population subjects born in the Netherlands between 1911 and 1949. Similarly, Glindmeyer *et al*. [9] reported an increase in height-specific vital capacity in a literature review of studies published between 1846 and 1975. Lak *et al*. [10] also found that Swedish 75-year-olds born in 1930 had higher peak expiratory flow than 75-year-olds born 1901–1902, after adjustment for several confounders. More recently, Allinson *et al*. [11] showed a height-independent increase of FEV_1_ and FVC with year of birth, analysing data from 10 cross-sectional European population-based studies of subjects born between 1884 and 1996.

Our results agree in general with these observations, showing an increase in mean lung function in Norwegian subjects born between 1894 and 1969. The increase of FEV_1_ and FVC was particularly evident for subjects born in the first part of the century, with only minor changes thereafter. The interpretation of these findings is, however, related to the reference equations used. Subjects born in the early 1900s are likely not very represented in the recently developed GLI-2012 equations. It was therefore possible to observe cohort effects in this particular subgroup. On the contrary, the absence of major cohort effects for subjects born approximately after 1935 might in part reflect a better fit of our sample with the GLI population in terms of years of birth. As GLI-2012 equations are derived from collated data, the same age might correspond to different years of birth. Cohort effects, therefore, if present, might have been included in the resulting equations.

While we cannot exclude the presence of some cohort effects in subjects born after 1935, it is also possible that the increase in lung function has indeed slowed in more recent years. This has also been suggested previously [11], where the authors, despite estimating homogeneous lung function changes with year of birth, observed that the increase appeared smaller among more recent birth cohorts. Moreover, they suggested that the cohort effects evidenced could lead one to progressively underestimate normal European lung function measured with GLI-2012 equations. Our results would agree with an underestimation of normal lung function for Norwegian subjects born approximately after 1935 but would show uncertainty as to whether this is determined by persistent cohort effects.

The FEV_1_ and FVC z-scores of healthy subjects born after 1935 in our sample are in line with a previous study investigating the fit of the GLI-2012 reference equations in the Norwegian population, showing slightly above-predicted values in Norwegian subjects, especially for females [20]. In that study, Norwegian adolescents showed better fit with GLI-2012 equations than adults. This might somewhat support the hypothesis of slowing cohort effects. As expected, FEV_1_ and FVC z-scores in our study were lower when examining changes in the total population, but the general birth cohort pattern was substantially unchanged.

As height is an important determinant of lung function, its significant increase is likely to largely explain the rising trend of FEV_1_ and FVC in our study. Smoking habits, education, prevalence of lung diseases and respiratory symptoms also proved to be significant contributors in our models. Other factors were, however, unexplored in our data, such as changes in childhood infections, healthcare quality, occupational and environmental exposures or nutrition. Future studies should also take such variables into consideration.

The use of relative lung function values limited the possibility to optimally investigate the contribution of height to the changes in spirometric measures in our study. We have, however, separately explored the trends of height with birth cohort and observed that its increase closely reflected the increase in FEV_1_ and FVC in the first part of the century. In more recent birth cohorts the trend of lung function was slightly more flattened than the trend of height. As previously mentioned, this likely reflects that in subjects born 1935–1969 the effect of height is mostly accounted for in the prediction equations. On the contrary, subjects born 1894–1935 are on average shorter, for a given age, than those included in the GLI population. This allowed a better visualisation of the increase in lung function attributable to height in this category.

With regard to the factors explaining changes in physical characteristics of the Norwegian population during the 20th century, many of these are probably related to economic growth. As discussed by Bore [21], economic factors can affect height *via* access to nutrients, work intensity and access to health services. Their study reported that between 1907 and 1977 the gross domestic product per capita in Norway increased by nearly seven times [21]. The Norwegian state has gradually introduced universal healthcare, free education, sick leave payment, and maternity and paternity leave alongside other welfare benefits. Numerous other changes occurred in Norwegian society over the course of the century, such as a rapid increase of the total population (from 2.2 million to about 4.5 million), general improvement in living conditions, transition from a predominantly agricultural to a predominantly industrial society, significant decline of mortality due to infections such as tuberculosis and dramatic reduction of infant mortality [22].

Our study examined data for subjects born until 1970, but several factors have changed since then. The mean height of Norwegian military recruits has not increased much since 1975 [23]. In contrast, from 1973 to today rates of daily smoking in Norway have declined substantially, both for males and females [24]. We thus need new studies of subjects born more recently to see how lung function trends have evolved. Immigration has also increased in the last decades in Norway, particularly from Eastern Europe and Asia, with immigrants and Norwegian-born to immigrant parents constituting respectively 13.8% and 3% of the total population in 2017, while corresponding figures in 1997 were 4.5% and <1% [25]. The effect of multi-ethnicity on normal lung function in Norway should also be a topic of further research.

When considering the variability of FEV_1_ and FVC between surveys, our results also suggest the presence of period effects. In case of perfect agreement with the selected reference equations, mean±sd z-scores of a healthy sample should be 0±1 [26]. In our study, FEV_1_ and FVC z-scores of healthy subjects had values close to zero in the three more recent surveys, but positive in the first one.

It is possible that our findings may be explained by the different spirometry quality criteria used in the different surveys. As increasingly stringent reproducibility criteria were applied over the study time (no fixed criteria, ECCS 1983 [16] and ATS 1994 [17]), this might have resulted in a relative overestimation of values in our earliest survey where fewer measurements were excluded. The different measurement conditions (ATPS *versus* BTPS) of the first survey might represent another explanation, as well as differences in the models of spirometry equipment.

The participation rate of the various surveys (clinical examination) also declined over time, in line with literature reports [27]. This might also have contributed to the observed period effects. Finally, it is possible that some of the differences in lung function between surveys might also reflect cohort effects, *i.e.*, the different distribution of years of birth in the various surveys might have determined a different degree of fit of the GLI-2012 equations.

Quanjer *et al*. [12] investigated period effects in a collated dataset of over 40 000 white healthy subjects and found no significant correlation between year of measurement and FEV_1_ or FVC z-scores. The apparent contrast with our findings might be explained by the periods of time in which the studies were performed. The data of their study were collected between 1978 and 2009, while our study was slightly earlier (1965–1999). When considering only the latest examinations (1988–1999), our results also do not show significant period effects. Quanjer *et al*. also explored the z-scores distribution of 30 different centres, while our population was more homogeneous. It is possible that certain minor trends in their study might have been obscured by the heterogeneity of the populations.

Period effects were also explored in some previous studies with inconsistent conclusions [8, 28–31]. While one study showed an increase in FEV_1_ in subsequent survey periods from 1965 to 1990 [8], others reported changes in lung function between surveys more similar to our findings [28–31]. All these studies were, however, performed on longitudinal data, which limits the comparability with our results.

The interpretation of lung function measurements in clinical practice relies on comparison with reference values derived from samples of healthy individuals. Several studies have explored them in Norway in the past [32–34]. Recently, multi-ethnic all-age reference equations have been developed, with the aim to allow more generalisable predictions across populations and promote the standardisation of the interpretation of lung function tests worldwide [19]. Period and cohort effects might affect reference standards, especially if reference populations are built over a large period of time. This might result in increased variation that should be considered when interpreting lung function results. Equations should be periodically reviewed and updated to minimise the impact of secular trends.

Our study has several strengths. We have examined both period and cohort effects in a large number of individuals that were randomly selected from the general population, with a very wide range in years of examination and years of birth. The geographical area of our study population was well-defined. Our analyses were adjusted for a large number of covariates and also performed in a healthy sub-sample. All the surveys were initiated and conducted by investigators affiliated with the same institution, which ensured uniformity in data collection and management.

Our study also has several limitations. First, we estimated period and cohort effects only as relative lung function changes, and not absolute ones. We explored the study of cohort effects in terms of absolute lung function, but the collinearity between age and year of birth in our models precluded accurate estimates. Age-period-cohort analysis might have represented an alternative methodological solution, but this approach was not suitable given the non-continuous nature of our period data. Second, the different spirometry methodologies adopted in the surveys represent a limitation for the analysis of cohort effects, which however could not have been avoided given the nature of the study. The differences in lung function attributable to period effects were, however, accounted for in the cohort effects analysis by including survey as a random (intercept) effect. This also explains the apparently contradictory finding for which period and cohort effects differ in direction. Third, while we adjusted our cohort effects models for period effects, our period effects analysis was only descriptive and not adjusted for cohort ones. Fourth, the harmonisation of qualitative variables, *e.g.*, subjectively reported respiratory symptoms and lung diseases, was suboptimal due to a sometimes imperfect correspondence between the questionnaire items in the various surveys. Finally, we lacked detailed information regarding occupational exposures and possibly other confounders.

Another issue that deserves to be mentioned is that the number of males in the study was higher than females. All analyses were performed in strata by sex, in order to address this limitation. The close agreement in both period and cohort effects between males and females, in our opinion, suggests that the findings are robust and not influenced by the size of the populations. Additionally, the distribution in terms of birth cohorts of the male population was not homogeneous, due to the majority of the subjects originating from a single large survey. We have considered the possibility that this might have affected the cohort effects results. We believe that the similarity of the pattern with females, which instead had a more homogeneous birth cohort distribution, reasonably excludes this possibility.

With regard to the periods of data collection, the years of measurement of the study presented a gap between 1970 and 1988. This did not allow us to investigate with continuity the impact of period effects from 1965 to 1999 (*i.e.*, to explore whether the changes in lung function after the first survey were more or less gradual). Despite the gap in data collection, years of birth were continuously represented from 1894 to 1969, both for males and females. We therefore do not think that the cohort effects conclusions are affected by the non-continuous data collection.

Although the participation rate of the surveys can be considered quite high, as in any survey there is a risk of non-participation bias. Moreover, the geographical area of our study was limited to Western Norway. A previous study has shown, however, only minor differences between spirometric values of healthy reference samples collected in different areas of Norway, one of which is included in the population of this study [20]. This indirectly supports a certain generalisability of the findings. Nonetheless, validation studies would be very important.

In conclusion, our study showed that FEV_1_ and FVC have increased in Norwegian males and females in the first half of the 20th century, as a result of cohort effects. Period effects seemed to have decreased from 1965 to 1999, likely reflecting more comparable methodology. Future studies in more recent generations would be useful to determine if secular trends in lung function persist.

## Supplementary material

10.1183/23120541.00302-2022.Supp1 **Please note:** supplementary material is not edited by the Editorial Office, and is uploaded as it has been supplied by the author.
Supplementary material 00302-2022.SUPPLEMENT
