# Supplementary material for: Period and cohort effects: consequences on spirometric lung function in Norway during the 20th century
Source: ERJ Open Res. 2022 Dec 12;8(4):00302-2022. doi: 10.1183/23120541.00302-2022 (PMC9835971; doi:10.1183/23120541.00302-2022)
Supplement: Supplementary file 1 [file 00302-2022.SUPPLEMENT.pdf]

SUPPLEMENTARY MATERIAL

Period and cohort effects: consequences on spirometric lung function in Norway during the 20th century

Lucia Cestelli, Ane Johannessen, Knut Stavem, Amund Gulsvik, Rune Nielsen

**TABLE S1.**	Main characteristics of the four surveys included in the study.	p.2
**TABLE S2.**	Prevalence of respiratory symptoms and lung diseases in the total population.	p.3
**FIGURE S1.**	Adjusted predictions of FEV_1 z-scores according to birth cohort in the total population.	p.4
**FIGURE S2.**	Adjusted predictions of FVC z-scores according to birth cohort in the total population.	p.5
**FIGURE S3.**	Adjusted predictions of height according to birth cohort in the total population.	p.6

TABLE S1. Main characteristics of the four surveys included in the study.				
	**Years**	**Geography**	**Objectives**	**Design**
**BRCDS**	1965–1970	Bergen, old city borders	To examine the manifestations of cardiovascular and pulmonary diseases.	Random sample of subjects born on the 3rd, 13th, and 23rd of each month between 1894–1943.
**PSWON**	1988–1990	Hordaland county and Sauda municipality	To examine the association between occupational exposures and spirometric airflow limitation.	All eligible males born 1944–1958.
**HCRHS**	1996–1997	Hordaland county	To examine the urban-rural gradient of obstructive lung diseases and the contribution of occupational exposure.<br>To develop lung function reference values.<br>To estimate the prevalence of risk factors like allergies and respiratory viruses.	Two-phased, stratified, random sample. First follow-up: all those who lived in Bergen and 11 surrounding municipalities.
**BD-HUSK**	1998–1999	Bergen municipality	To evaluate the applicability of the adrenergic bronchodilator test and to estimate prevalence and predictors of a positive bronchodilatation response.	Follow-up of the Hordaland Homocystein Study population. Random sample of ten sex-birth year strata, of all subjects born 1925–1927 and 1950–1951.

BCRDS= Bergen Chronic Respiratory Disease Survey, PSWON= Pneumoconiosis Survey of Western Norway, HCRHS= Hordaland County Respiratory Health Survey, BD-HUSK= Bronchodilatation Survey of the Hordaland Health Study

TABLE S2. Prevalence of respiratory symptoms and lung diseases in the total population.

	BCRDS	PSWON	HCRHS	BD-HUSK	Total
**Males**					
Breathlessness walking uphill and/or climbing two flights of stairs	598 (24)	3431 (13)	180 (22)	245 (22)	4454 (15)
Attacks of breathlessness	38 (2)	2574 (10)	84 (10)	69 (6)	2765 (9)
Cough morning	246 (10)	2560 (10)	209 (26)	300 (27)	3315 (11)
Cough chronic	189 (8)	1386 (6)	100 (12)	118 (11)	1793 (6)
At least one respiratory symptom	737 (29)	6654 (26)	344 (42)	475 (43)	8210 (27)
Lung diseases	160 (6)	4212 (16)	147 (18)	274 (25)	4793 (16)
**Females**					
Breathlessness walking uphill and/or climbing two flights of stairs	1164 (38)	-	357 (30)	397 (24)	1918 (33)
Attacks of breathlessness	72 (2)	-	130 (11)	110 (7)	312 (5)
Cough morning	110 (4)	-	307 (26)	382 (23)	799 (13)
Cough chronic	93 (3)	-	150 (13)	189 (11)	432 (7)
At least one respiratory symptom	1210 (39)	-	556 (47)	688 (41)	2454 (41)
Lung diseases	147 (5)	-	313 (27)	426 (26)	886 (15)

Variables are presented as number (%).
BCRDS= Bergen Chronic Respiratory Disease Survey (1965–1970), PSWON= Pneumoconiosis Survey of Western Norway (1988–1990), HCRHS= Hordaland County Respiratory Health Survey (1996–1997), BD-HUSK= Bronchodilatation Survey of the Hordaland Health Study (1998–1999).

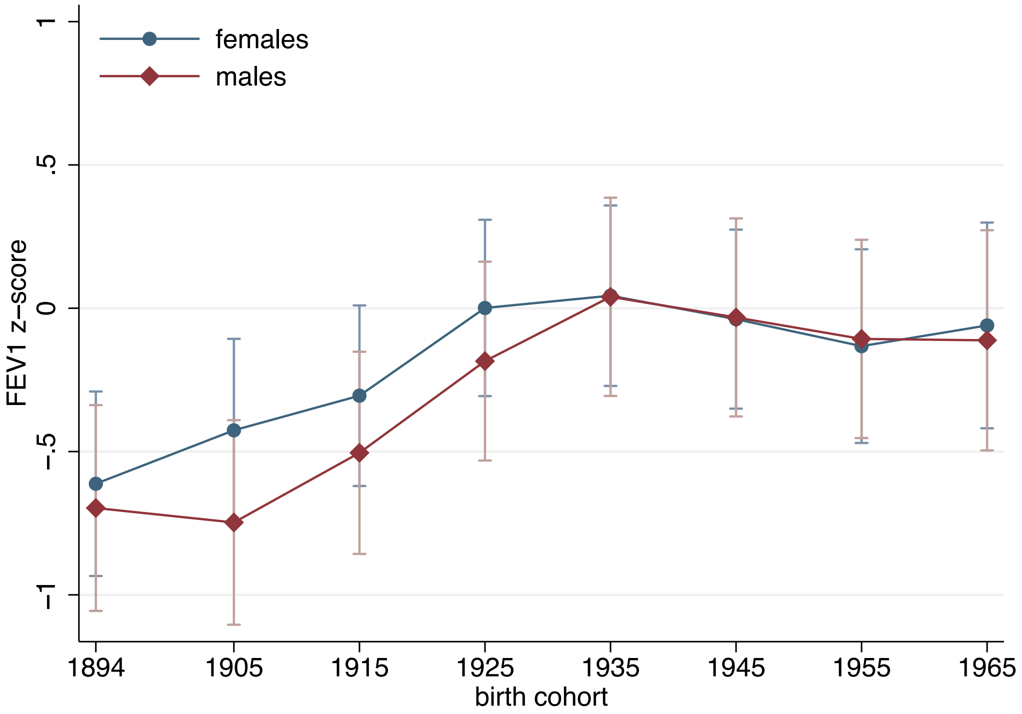

FIGURE S1. Adjusted predictions of FEV_1 z-scores according to birth cohort in the total population. *Z*-scores calculated according to GLI-2012 equations. C.I. 95%.

Birth cohorts: 1894–1904, 1905–1914, 1915–1924, 1925–1934, 1935–1944, 1945–1954, 1955–1964, 1965–1969.

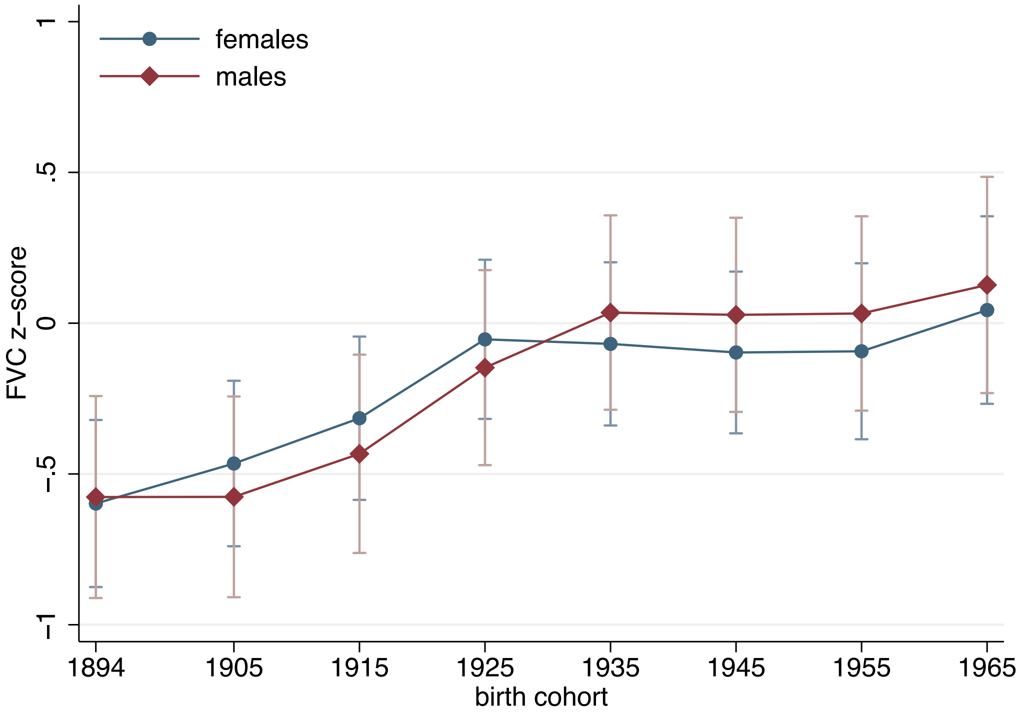

FIGURE S2. Adjusted predictions of FVC z-scores according to birth cohort in the total population. Z-scores calculated according to GLI-2012 equations. C.I. 95%.

Birth cohorts: 1894–1904, 1905–1914, 1915–1924, 1925–1934, 1935–1944, 1945–1954, 1955–1964, 1965–1969.

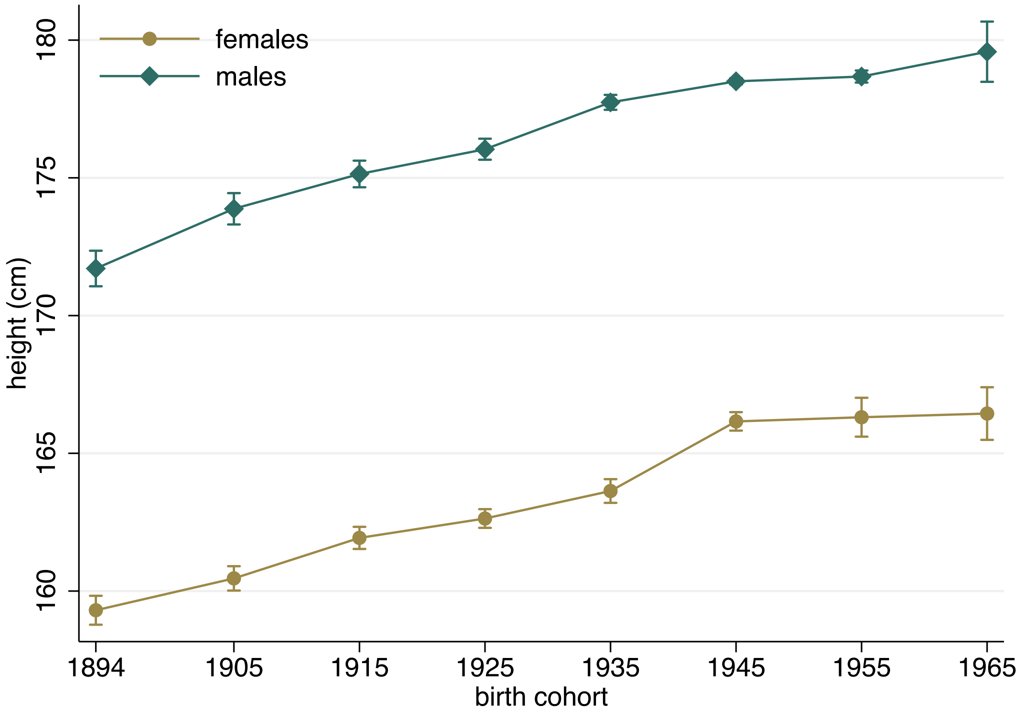

FIGURE S3. Adjusted predictions of height according to birth cohort in the total population, for 45-year-old males and females. C.I. 95%.

Birth cohorts: 1894–1904, 1905–1914, 1915–1924, 1925–1934, 1935–1944, 1945–1954, 1955–1964, 1965–1969.
